# *SLC7A8* coding for LAT2 is associated with early disease progression in osteosarcoma and transports doxorubicin

**DOI:** 10.3389/fphar.2022.1042989

**Published:** 2022-11-09

**Authors:** Evelien G. E. Hurkmans, Jan B. Koenderink, Jeroen J. M. W. van den Heuvel, Yvonne M. H. Versleijen-Jonkers, Melissa H. S. Hillebrandt-Roeffen, Johanne M. Groothuismink, Hanneke I. Vos, Winette T. A. van der Graaf, Uta Flucke, Grigor Muradjan, Hendrik W. B. Schreuder, Melanie M. Hagleitner, Han G. Brunner, Hans Gelderblom, Anne-Marie Cleton-Jansen, Henk-Jan Guchelaar, Eveline S. J. M. de Bont, Daan J. Touw, G. Jan Nijhoff, Leontien C. M. Kremer, Huib Caron, Rachael Windsor, Ana Patiño-García, Anna González-Neira, Federica Saletta, Geoff McCowage, Sumanth Nagabushan, Daniel Catchpoole, D. Maroeska W. M. te Loo, Marieke J. H. Coenen

**Affiliations:** ^1^ Department of Human Genetics, Radboud University Medical Center, Nijmegen, Netherlands; ^2^ Department of Pharmacology and Toxicology, Radboud University Medical Center, Nijmegen, Netherlands; ^3^ Department of Medical Oncology, Radboud University Medical Center, Nijmegen, Netherlands; ^4^ Department of Medical Oncology, Netherlands Cancer Institute, Amsterdam, Netherlands; ^5^ Department of Pathology, Radboud University Medical Center, Nijmegen, Netherlands; ^6^ Department of Orthopedics, Radboud University Medical Center, Nijmegen, Netherlands; ^7^ Princess Maxima Center for Pediatric Oncology, Utrecht, Netherlands; ^8^ Department of Medical Oncology, Leiden University Medical Center, Leiden, Netherlands; ^9^ Department of Pathology, Leiden University Medical Center, Leiden, Netherlands; ^10^ Department of Clinical Pharmacy & Toxicology, Leiden University Medical Center, Leiden, Netherlands; ^11^ Department of Pediatrics, Beatrix Children’s Hospital, University Medical Center Groningen, Groningen, Netherlands; ^12^ Department of Clinical Pharmacy and Pharmacology, University Medical Center Groningen, Groningen, Netherlands; ^13^ Department of Pediatrics, Amsterdam University Medical Centers, Emma Children’s Hospital, Amsterdam, Netherlands; ^14^ Pediatric & Adolescent Division, University College London Hospitals NHS Foundation Trust, London, United Kingdom; ^15^ Department of Pediatrics, Clínica Universidad de Navarra, Solid Tumor Program, CIMA, Pamplona, Spain; ^16^ Human Genotyping Unit-CeGen, Spanish National Cancer Research Centre (CNIO), Madrid, Spain; ^17^ Children’s Cancer Research Unit, The Children’s Hospital at Westmead, Sydney, NSW, Australia; ^18^ Cancer Centre for Children, The Children’s Hospital at Westmead, Sydney, NSW, Australia; ^19^ Discipline of Child and Adolescent Health, University of Sydney, Sydney, NSW, Australia; ^20^ Department of Pediatrics, Amalia Children’s Hospital, Radboud University Medical Center, Nijmegen, Netherlands

**Keywords:** osteosarcoma, pharmacogenetics, doxorubicin, L-type amino acid transporter 2, early disease progression

## Abstract

**Background:** Despite (neo) adjuvant chemotherapy with cisplatin, doxorubicin and methotrexate, some patients with primary osteosarcoma progress during first-line systemic treatment and have a poor prognosis. In this study, we investigated whether patients with early disease progression (EDP), are characterized by a distinctive pharmacogenetic profile.

**Methods and Findings:** Germline DNA from 287 Dutch high-grade osteosarcoma patients was genotyped using the DMET Plus array (containing 1,936 genetic markers in 231 drug metabolism and transporter genes). Associations between genetic variants and EDP were assessed using logistic regression models and associated variants (*p* <0.05) were validated in independent cohorts of 146 (Spain and United Kingdom) and 28 patients (Australia). In the association analyses, EDP was significantly associated with an *SLC7A8* locus and was independently validated (meta-analysis validation cohorts: OR 0.19 [0.06–0.55], *p* = 0.002). The functional relevance of the top hits was explored by immunohistochemistry staining and an *in vitro* transport models. *SLC7A8* encodes for the L-type amino acid transporter 2 (LAT2). Transport assays in HEK293 cells overexpressing LAT2 showed that doxorubicin, but not cisplatin and methotrexate, is a substrate for LAT2 (*p* < 0.0001). Finally, *SLC7A8* mRNA expression analysis and LAT2 immunohistochemistry of osteosarcoma tissue showed that the lack of LAT2 expression is a prognostic factor of poor prognosis and reduced overall survival in patients without metastases (*p* = 0.0099 and *p* = 0.14, resp.).

**Conclusion:** This study identified a novel locus in *SLC7A8* to be associated with EDP in osteosarcoma. Functional studies indicate LAT2-mediates uptake of doxorubicin, which could give new opportunities to personalize treatment of osteosarcoma patients.

## Introduction

Osteosarcoma is a malignant bone tumor that mainly affects children and adolescents. Although the number of people affected by osteosarcoma is low (worldwide 3-4 patients per million), the disease is ranked as one of the most frequent causes of cancer-related death in young patients (Siegel et al., 2019). The disease has a great impact on the patient’s life, as treatment requires an intensive combination of chemotherapy, often disabling surgery, and prolonged periods of rehabilitation. Despite this harsh treatment regimen, some patients fail to respond, showing no response or even tumor growth during primary treatment (early disease progression—EDP). Recognition of patients that do not benefit from current chemotherapy schedules at an early phase in treatment is therefore important.

Although the genetics and biology of the tumor are likely to contribute to the heterogeneous response to treatment, we postulate that germline variants in drug metabolizing enzymes or transporters might also contribute to this observed heterogeneity. Pharmacogenetics holds the promise to identify germline genetic variants predictive of drug response in individual patients. Most studies aimed at identifying germline genetic variants predictive of treatment outcome in osteosarcoma have considered survival as the main clinical endpoint. However, patients with EDP, who may have a distinctive pharmacogenetic profile, have not been widely studied as a subgroup. The few studies comparing complete or partial responders investigated only a few candidate genes involved in DNA repair (*CCNH, ERCC1*/*2*/*5*/*6*, *MMS19L* and *XPC*) and *GSTP1,* a gene involved in detoxification of exogenous and endogenous compounds (Yang et al., 2012; Bai et al., 2013; Sun et al., 2013; Liu et al., 2015). Significant associations of genetic variants in *ERCC2*/*5*, *MMS19L*, *XPG* and *GSTP1* with clinical response have been identified. However, validation in additional samples is necessary to confirm these associations as the results are conflicting (Bai et al., 2013; Sun et al., 2013; Zhao et al., 2013).

We performed a screening of 1,936 genetic variants in 231 drug metabolism and transporter genes in a group of 207 osteosarcoma patients with the objective of discovering a relationship with suboptimal drug response during the first-line treatment, followed by validation in two independent cohorts of 57 and 24 patients. Finally, we studied the top hit gene by performing functional assays to elucidate the biological mechanism behind the association.

## Methods

### Patient cohorts

A discovery cohort of 287 osteosarcoma patients was retrospectively collected at the Radboud university medical center (Nijmegen), the University Medical Center of Groningen (Groningen), Leiden University Medical Center (Leiden) and the Emma Children’s Hospital/Academic Medical Center (Amsterdam), Netherlands. All patients were treated between 1978 and 2013 and the clinical data were retrospectively collected from medical records. Eligibility criteria were: histological diagnosis of primary high-grade osteosarcoma with or without metastatic disease, age ≤ 45 years, treatment with cisplatin and doxorubicin-based chemotherapy (also neoadjuvant), and self-reported Caucasian ethnicity. Patients were treated either according to institutional standard therapy consisting of cisplatin (maximum cumulative dose 600 mg/m^2^) and doxorubicin (maximum cumulative dose 450 mg/m^2^), or according to the standard schedule as given in the EURAMOS-1 trial, which consisted of cisplatin (480 mg/m^2^)/doxorubicin (450 mg/m^2^) and additionally high-dose methotrexate (MTX; 144 g/m^2^), with or without additional ifosfamide/etoposide or interferon-α (Whelan et al., 2015).

A combined cohort of 146 high-grade osteosarcoma patients treated with cisplatin and doxorubicin-based chemotherapy from Spain (*N* = 95) and England (*N* = 51) was used for independent validation of the positive findings in the discovery cohort (Caronia et al., 2011; Windsor et al., 2012). Information on treatment has been reported previously (Caronia et al., 2011; Windsor et al., 2012). In a second validation phase, a cohort of 28 high-grade osteosarcoma patients, treated with cisplatin and doxorubicin-based chemotherapy from Australia (Sydney Children’s Tumour Bank Network), was included. Patients were treated with a cisplatin cumulative dose of 480 mg/m^2^ or 600 mg/m^2^ and doxorubicin cumulative dose of 450 mg/m^2^. The same inclusion criteria as used in the discovery cohort were applied, with the exception of ethnicity.

The study was approved by the Institutional review board of the Radboud university medical center, and approval for inclusion of patients in other institutions was obtained from the corresponding institutional ethics committees. All patients and/or parents provided written informed consent.

### Response definition

The clinical (radiological) response to treatment was based on imaging results (CT/MRI/X-ray) reviewed by local expert radiologists. EDP was defined as: 1) growth of the primary tumor (>20%) and/or metastases (>20%), or development of new lesions, in the time from start of primary treatment until 3 months after end of adjuvant chemotherapy or end of first-line treatment in case of primary metastatic disease, and/or 2) inadequacy to reach complete remission at the end of (surgical and chemotherapeutic) therapy for primary localized or primary metastatic osteosarcoma. The opposite extremes, patients showing an adequate drug response with no signs of relapse were considered as patients with no disease progression. Thus, patients with recurrent disease, defined as local or distant relapse later than 3 months after end of primary treatment to end of follow-up, were excluded from the analysis.

### Genotyping methods

For the discovery cohort, germline DNA was isolated from blood using the QIAamp DNA Blood Midi kit (Qiagen, Venlo, Netherlands), or from saliva using the Oragene saliva collection kit (DNA Genotek, Kanata, Ontario, Canada) according to the manufacturer’s protocols. From patients who had died before inclusion in this pharmacogenetic study, DNA was isolated from normal formalin-fixed, paraffin-embedded tissue as described previously (Hagleitner et al., 2011a; Vos et al., 2015). However, for 16 patients of the initial Dutch patient cohort, DNA yield isolated from formalin-fixed, paraffin-embedded tissue was too low for array genotyping. These patients were deceased with often progressive disease. Therefore, the exclusion of these patients was non-random, possibly inducing bias. The limited available DNA was used to manually genotype the top single nucleotide polymorphisms from the study to assess the effect of exclusion on the main findings. The DNA samples from all other patients were genotyped for 1,936 genetic variants using the Affymetrix DMET Plus array according to the manufacturer’s instructions (Affymetrix United Kingdom Ltd., High Wycombe, United Kingdom). Genotypes were calculated with DMET console software 1.3 using the Dynamic Genotype Boundaries version 2 algorithm. Variants were excluded from analysis if the genotype cluster plots were considered unreliable, being plots with genotype calling showing merged clusters without distinct cluster boundaries. Additional stringent evaluation of the genotype clustering in combination with expected genotype frequencies was carried out for variants significant in association analysis. Quality control was carried out on the total cohort of 372 genotyped patients. Samples and variants were excluded if call rates <0.9, minor allele frequency <0.01 and/or deviating from Hardy-Weinberg equilibrium (*p*-value <0.0001). The five copy number variants, 46 X-chromosomal variants and one tri-allelic variant present on the array were not included in the analyses.

Isolation of germline DNA in the validation cohort has been previously reported (Caronia et al., 2011; Windsor et al., 2012); from the Australian validation cohort germline DNA was isolated from blood using the QIAamp DNA Blood Mini kit (Qiagen) according to the manufacturer’s instructions. Genotyping of the validation cohort was performed for seven of the ten significant variants in the discovery cohort, excluding three variants that were in linkage disequilibrium with any of the seven variants (based on r^2^ ≥ 0.80) and that had higher *p*-values than their linked variants. Genotyping of the second validation cohort was subsequently performed for the five variants that were significant in the first validation stage and that showed the same direction of effect in the discovery and validation cohorts. KASP-On-Demand (KOD) assays were used for *CYP4F12* rs688755, *SLC22A5* rs274548, *FMO6* rs7886938 and *SLC7A8* rs8013529; KASP-By-Design assays were used for *CYP8B1* rs6771233, *SLC22A2* rs316003, and *SLC7A8* rs1884545, all according to the manufacturer’s protocol (LGC Genomics, Hoddesdon, United Kingdom). Fluorescence was measured with a 7500FAST Real-Time PCR System (ThermoFisher, Nieuwegein, Netherlands). Genotypes were scored using the algorithm and software (v2.0.6) supplied by ThermoFisher. Blanks (3%) as well as duplicates between plates were included as quality controls for genotyping.

### Statistical analysis

Statistical differences in demographic data between patients with EDP and control patients were assessed by the Fisher exact, Pearson chi-square or Mann-Whitney U tests as appropriate using SPSS v22 (SPSS Inc., Chicago, Ill, United States). To assess the effect of a genetic variant on EDP, the data were dichotomized to EDP yes/no Associations between genetic variants and EDP were assessed by multivariable logistic regression analysis in PLINK using the command-logistic (additive model) (PLINK v1.07) (Purcell et al., 2007). For genetic variants significantly associated with EDP in the discovery cohort, we assessed potential associations with two other clinical endpoints: recurrent disease (using PLINK), and 5-year DFS (time interval from diagnosis to either progression or recurrence) using Cox proportional hazards models in SPSS. These variants were excluded from subsequent analysis, to filter out those variants that were not specific for the inadequate drug response observed in EDP patients. Reported *p*-values are two-sided and are considered statistically significant if < 0.05 in the genetic analyses (<0.05 for selection of clinical covariables). No correction for multiple testing was performed because of the exploratory nature of the study. Meta-analysis of the association analysis results of the discovery and validation cohorts, and of all three cohorts including the second validation cohort, was performed using a fixed effects or random effects (in case of large heterogeneity: I^2^ > 50) model in PLINK.

### Cell culture and transduction

HEK293 cells were cultured in Dulbecco’s Modified Eagle Medium (DMEM) containing GlutaMAX with 10% (*v/v*) Fetal Bovine Serum (FBS) at 37°C, 5% CO_2_ until 70–80% confluence. Cells were diluted 1:5 and ∼300,000 cells per well were seeded into Poly-D-lysine coated 24-wells plates. Twenty-four hours after seeding, cells were transduced with 60–90 µL of recombinant baculoviruses and sodium butyrate (3 mM) was added to a final volume of 600 µL. These baculoviruses were modified to induce protein overexpression in HEK293 cells as previously described (El-Sheikh et al., 2007). Briefly, cDNA of the control, LAT2 or 4F2 was cloned downstream of a CMV promotor in the baculoviruses using a Bac-to-Bac gateway system (Invitrogen, Breda, Netherlands). The sequences of the cDNAs are equal to the reference genome (GRCh37/hg19) without genetic variants. During transduction, the baculoviruses induced expression of EYFP (negative control), LAT2 or 4F2. For expressing both LAT2 and 4F2, cells were transduced with both baculoviruses. After transduction, cells were incubated for 48–72 h at 37°C, 5% CO_2_.

### Transport assay

After HEK293 cell transduction, the transport assay was performed in a 24-wells plate. Culture medium was removed and cells were washed with Na^+^-free buffer (37°C) containing 125 mM choline chloride, 4.8 mM KCl, 1.2 mM MgSO_4_, 1.2 mM KH_2_PO4, 1.3 mM CaCl_2_, 5.6 mM glucose and 25 mM HEPES at pH 7.4, according to Khunweeraphong *et al.* (Khunweeraphong et al., 2012). Thereafter, cells were exposed to a radiolabeled compound (^3^H-methotrexate or ^3^H-alanine) or an unlabeled compound (cisplatin or doxorubicin) diluted in Na^+^-Free HBSS-HEPES buffer at 37°C. When studying inhibition, cells were exposed to a mixture of a substrate and an inhibitor. ^3^H-L-alanine and BCH (2-Aminobicyclo (2.2.1) heptane-2-carboxylic acid) were used as known substrate and inhibitor, respectively, to validate the model. Exposure time and concentrations are specified per experiment in the results section. After incubation, cells were washed with ice cold Na^+^-free buffer containing 0.5% BSA (*m/v*) (4°C) and with Na^+^-free buffer only (4°C). Ultimately, cells were lysed in 1-0.1 M NaOH or 0.1% Triton X-100.

To determine uptake of radiolabeled substrates, cell lysates were mixed with 4 ml OPTI-FLUOR (PerkinElmer, Waltham, MA, United States) and analyzed with the Hidex automatic TDCR liquid scintillation counter (Turku, Finland). Doxorubicin uptake was measured in lysed cells with 0.1% Triton X-100 by determining the fluorescence of doxorubicin using the Victor X3 multimode plate reader at an excitation and emission wavelength of 485 nm and 590 nm, respectively (PerkinElmer Nederland B.V., Groningen, Netherlands). Cisplatin uptake concentrations were determined using inductively coupled plasma mass spectrometry. For this, a Thermo Scientific (Waltham, Massachusetts, United States) iCAP TQ mass spectrometer was used (in SQ modus). The system operated under the standard conditions, briefly: Rf power 1550 W, argon gas flow rates: cooling 14 L/min, auxiliary 0.8 L/min, nebulizer 1.03 L/min, sampler, skimmer: nickel, spray chamber: cyclone, 3°C, torch: quartz, nebulizer: concentric, data acquisition: masses 89, 195, sweeps 10, dwell time 100 ms. The calibration standards were prepared from commercially available stock solutions. The platinum standard (1,000 mg/L) was supplied by VWR (Leicestershire, England). Yttrium, used as the internal standard, was prepared from a 1,000 mg/L solution obtained from Merck (Darmstadt, Germany). Measurements were performed in a matrix of 0.1 M NaOH, which is consistent with the lysis buffer of cells in the transport experiment (Merck—Darmstadt, Germany).

The protein concentration was determined (Bio-Rad Laboratories, Lunteren, Netherlands) and measured using a Benchmark Plus plate reader (595 nm). The transport assays were corrected for protein content in the wells. The mean ± SD of three experiments with cells transduced by independently produced baculoviruses was plotted with GraphPad Prism version 8. Significant uptake was determined using a one-way ANOVA, comparing conditions to control, with Dunnett’s post-test. To determine if inhibition was significant, relative substrate uptake in the presence of inhibitor was compared to a solvent only control using an independent *t*-test.

### SDS-page and western blot

LAT2 protein expression was determined with SDS-PAGE and western blotting. HEK293 cells were transduced according to the same protocol as in preparation for the transport assays, but in a T75 flask. Subsequently, cells were lysed and the total membrane fraction (20000 g) was run on a SDS-Page and transferred to a Nitrocellulose membrane. LAT2 was visualized with the Odyssey (LI-COR) and the SLC7A8 mouse monoclonal antibody, clone UMAB70 (OriGene, Analog No: UM570058)

### Immunohistochemistry

Immunohistochemistry staining on 4 µm thick slides from eight osteosarcoma tissue micro arrays (one from the Australian cohort and seven from the Dutch cohort), with one or two 2.0 mm cores per tumor samples from representative tumor areas, was performed to assess LAT2 protein expression and p-mTOR expression to assess possible LAT2 mediated mTOR phosphorylation. Pancreas and kidney served as positive controls for LAT2 and p-mTOR, respectively. Sections were deparaffinized in xylene and rehydrated through a graded ethanol into water series. Antigen retrieval was performed by heating the slides in citrate buffer, pH6 for 10 min (p-mTOR) at 100°C or heating the slides in the 2100-Retriever (Diagnostic Technology, Belrose, Australia) (LAT2). Endogenous peroxidase activity was blocked with 3% H_2_O_2_ in distilled water for 10 min at room temperature. Subsequently, sections were incubated with monoclonal mouse anti-LAT2 antibody (1:50, UMAB70 antibody, OriGene Technologies, Inc., Rockville, United States) or monoclonal rabbit anti-phospho-mTOR (Ser2448) (1:50, #2976, Cell Signaling Technology, Danvers, United States) in antibody diluent in a humidified chamber overnight at 4°C. Next, tissue sections were incubated with Poly-HRP-GAMs/Rb IgG (ImmunoLogic, Duiven, Netherlands) in EnVision^™^ FLEX Wash Buffer (Dako, Agilent Technologies, Santa Clara, CA, United States) (1:1) for 30 min at room temperature. Antibody binding was visualized using the EnVision^™^ FLEX Substrate Working Solution (Dako) for 10 min at room temperature. Finally, slides were counterstained with haematoxylin, dehydrated and coverslipped. Only tissue samples from diagnostic biopsies, that were not exposed to chemotherapy, were analyzed. Tissue micro arrays were scored by two independent observers on percentage of cells with expression (none, <10%, 10–50% or >50%) and intensity of expression (0, + or ++). Differences between observers were resolved by consensus. Eventually, expression data were dichotomized and tissues with expression ≥10% of cells were considered positive for LAT2 or p-mTOR expression.

### SLC7A8 mRNA expression analysis

*SLC7A8* mRNA expression data of osteosarcoma tissue of a previous study by Kuijjer et al. was accessed through the ‘R2: Genomics Analysis and Visualization Platform’ (http://r2.amc.nl) (Kuijjer et al., 2012). The R2 online interface was utilized for the visualization of the data in a Kaplan Meier curve, with subsequent Log-Rank test for significance. The algorithm in this online interface scanned for a cutoff between “high” and “low” expression that yields the lowest *p*-value in the Log-Rank test in this cohort.

## Results

### Patient population

The genetic study was carried out following a three-stage design, including a discovery cohort, and two independent validation cohorts. Of the 287 eligible patients in the discovery cohort, four patients were excluded based on a genotype call rate lower than 0.9, leaving 283 patients for analysis (Supplementary Figure S1). From the first validation cohort, all 146 patients were successfully genotyped, while from the second, one of the 28 patients was excluded based on genotyping failure for all variants, leaving 27 patients for analysis. The patient characteristics of the three cohorts are provided in Table 1. EDP was observed in 13.8%, 12.3% and 18.5% of patients in the discovery, first and second validation cohorts, respectively.

**TABLE 1:** Clinical characteristics of the osteosarcoma patients of the discovery and validation cohorts.

	Discovery cohort		Validation cohort		Second validation cohort	
	Progression (*N* = 39)	No progression (*N* = 168)	Progression (*N* = 18)	No progression (*N* = 93)	Progression (*N* = 5)	No progression (*N* = 19)
Age at diagnosis, median (range)	17.2 (6.8–44.7)	15.2 (3.4–45.8)	15.4 (1.9–37.0)	15.0 (6.1–39.0)	15.3 (8.7–16.8)	14.2 (1.1–16.3)
Male sex, *n* (%)	29 (74.4%)	81 (48.2%)	11 (61.1%)	47 (50.5%)	4 (80.0%)	7 (36.8%)
Axial tumor, *n* (%)	4 (10.3%)	5 (2.98%)	1 (5.6%)	2 (2.2%)	1 (20.0%)	0 (0%)
Primary metastases, *n* (%)	16 (41.0%)	16 (9.52%)	9 (50.0%)	8 (8.6%)	3 (60.0%)	1 (5.3%)
Cumulative dose (mg/m^2^)						
Cisplatin^a^, median (range)	400 (100–600)	480 (200–720)	354 (175–1,064)	474 (0–1,038)	480 (480–600)	480 (480–600)
Doxorubicin, median (range)	450 (150–450)	450 (150–455)	242 (143–950)	385 (90–623)	450 (450–450)	450 (450–450)
Methotrexate treatment^b^, *n* (%)	27 (69.2%)	90 (53.6%)	16 (100%)	86 (96.6%)	5 (100%)	15 (78.9%)
Poor histologic response^c^, *n* (%)	28 (82.4%)	79 (49.4%)	10 (62.5%)	37 (41.6%)	4 (100%)	4 (44.4%)
5-year overall survival	26.8%	100%	33.3%	98.9%	30.0%	100%

Patients with recurrent disease are not included in the table: discovery cohort *N* = 76, validation cohort *N* = 35, second validation cohort *N* = 3.

^a^ Number of patients with cumulative dose of cisplatin data available; discovery cohort: progression *N* = 39, controls *N* = 165; additional cohort: all patients.

^b^ Number of patients with methotrexate treatment data available; discovery cohort: all patients; validation cohort: progression *N* = 16, controls *N*= 89; second validation cohort: progression *N* = 5, controls *N* = 19.

^c^ Number of patients with histologic response data available; discovery cohort: progression *N* = 34, controls *N* = 160; validation cohort: progression *N* = 16, controls *N* = 89; second validation cohort: progression *N* = 4, recurrence *N* = 2, controls *N*= 9.

In the discovery cohort, of all clinical variables included in Table 1, male gender (*p* = 0.003), the presence of primary metastases (*p* <0.001), and poor histologic response (*p* <0.001) were significantly associated with the occurrence of EDP. Therefore, these were included as clinical covariates in the genetic analyses, with the exception of the histologic response. Histologic response and progression are both a reflection of the response to chemotherapy, inclusion of the histologic response as covariate would unintentionally remove variation between EDP and patients without early disease progression and would therefore result in overcorrection. Despite that the association with increased age at diagnosis was not significant (*p* = 0.058), age was included as a covariate since it was previously identified as a prognostic factor in osteosarcoma (Hagleitner et al., 2011b). Supplementary Table S1 shows demographic data of the additional cohort of patients that was non-randomly excluded from the initial association analysis due to limited availability of DNA.

### Association analyses in the discovery cohort

**FIGURE 1:**
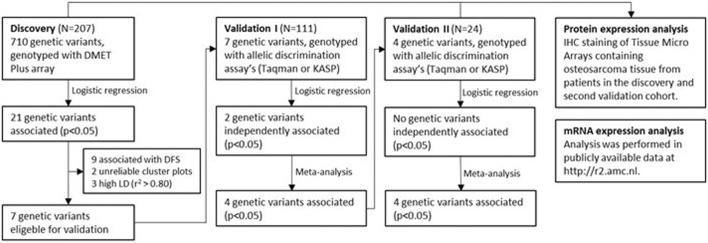
Flowchart of discovery and validation phase of this study. Details on quality control of genetic data are shown in Supplementary Figure S1. DMET, drug metabolizing enzymes and transporters; DFS, disease free survival; LD, linkage disequilibrium; IHC, immunohistochemistry.

Of the 1,884 variants included in the quality control, 90 variants were excluded based on unreliable cluster plots, 28 variants were excluded because of genotype call rates of <0.9 and 1,056 variants because of a minor allele frequency of <0.01. All remaining variants were in Hardy-Weinberg equilibrium. After quality control, 710 variants were included in the analysis of the discovery cohort (Figure 1, Supplementary Figure S1). In multivariable logistic regression analysis, comparing patients with EDP to those without EDP, including sex, age at diagnosis, and the presence of primary metastases as covariates, 12 genetic variants were significantly associated with EDP, after filtering of variants also associated with recurrent disease or 5-year disease free survival (DFS). The genotype cluster plot of two of these variants, *CYP2B6* rs2279341 and *CYP2D6* rs1058164, were considered unreliable after additional stringent evaluation of the clustering combined with expected genotype frequencies, leading to 10 remaining variants in 6 genes (Table 2, Supplementary Table S2).

**TABLE 2:** Results of the association analysis in the discovery and validation cohorts. In the discovery cohort, 710 genetic variants in drug metabolizing enzymes and transporters were assessed for their association with early disease progression (EDP) in patients with osteosarcoma. Variants with a significant association were manually genotyped and analyzed in the validation cohorts. The results were combined in meta-analyses.

				Discovery				Validation						Second validation		
								Meta-analysis			Meta-analysis			Meta-analysis overall		
SNP	Gene	Chromosome	MAF	Minor allele	OR	95% CI	*p*-value	OR	95% CI	*p*-value	OR	95% CI	*p*-value	OR	95% CI	*p*-value
rs8013529	*SLC7A8*	14	0.128	C	0.23	0.06–0.81	0.023	0.05	0.005–0.63	0.020	0.16	0.05–0.52	0.002	0.19	0.06–0.55	0.002
rs1884545	*SLC7A8*	14	0.127	T	0.22	0.06–0.78	0.019	0.05	0.005–0.63	0.020	0.16	0.05–0.50	0.002	0.22	0.07–0.63	0.005
rs6771233	*CYP8B1*	3	0.332	A	1.95	1.08–3.54	0.027	1.70	0.78–3.72	0.184	1.86	1.16–2.98	0.010	1.91	1.20–3.05	0.006
rs6774801	*CYP8B1*	3	0.330	A	1.95	1.08–3.54	0.027	NA	NA	NA	NA	NA	NA	NA	NA	NA
rs316003	*SLC22A2*	6	0.205	G	0.44	0.20–0.97	0.042	0.58	0.20–1.69	0.318	0.48	0.25–0.92	0.026	0.49	0.26–0.91	0.023
rs274548	*SLC22A5*	5	0.146	T	2.13	1.01–4.48	0.048	1.75	0.59–5.18	0.311	2.00	1.08–3.69	0.027	1.94	1.08–3.52	0.028
rs7886938	*FMO6*	1	0.170	A	2.14	1.06–4.32	0.033	0.83	0.22–3.17	0.788	1.75	0.94–3.25	0.078	NA	NA	NA
rs7889839	*FMO6*	1	0.170	G	2.14	1.06–4.32	0.033	NA	NA	NA	NA	NA	NA	NA	NA	NA
rs688755^a^	*CYP4F12*	19	0.228	C	2.06	1.07–3.98	0.031	0.73	0.29–1.83	0.499	1.29	0.47–3.57	0.623	NA	NA	NA
rs593421	*CYP4F12*	19	0.221	C	2.02	1.06–3.84	0.033	NA	NA	NA	NA	NA	NA	NA	NA	NA

OR, odds ratio; 95% CI, 95% confidence interval; NA, not analyzed.

OR, and 95% CI, are reported for the minor allele, i.e. an OR<1 indicates risk of progression for the major allele.

Variants within *CYP4F12*, *CYP8B1* and *FMO6* are in linkage disequilibrium (LD) with r^2^ > 0.80. LD, statistic in this cohort for variants in *SLC7A8* are r^2^ = 0.78 and D’ = 0.89.

^a^ I^2^ > 50. SNP: Single Nucleotide Polymorphism, MAF: Minor Allele Frequency, OR: Odds Ratio, CI: Confidence Interval.

### Validation results

All variants for validation were in Hardy-Weinberg equilibrium (*p* >0.05) and showed an average call rate of 0.98. Upon multivariable logistic regression analysis, two of the seven genetic variants showed a significant association with EDP (Table 2). For these variants, rs8013529 and rs1884545 located in the Solute Carrier Family 7 (Amino Acid Transporter Light Chain, L System) Member 8 (*SLC7A8*) gene, a protective effect was observed for T allele carriers in case of rs1884545 (*p* = 0.020) or C allele carriers forrs8013529. The effect remained significant with addition of inclusion site as covariate (*p* = 0.018).

### Meta-analysis

A meta-analysis was performed of the seven variants. Two variants in the *SLC7A8* locus independently validated and the effect further reinforces when combined in a meta-analysis*.* The rs1884545 variant showed a significant protective effect in patients with EDP with an odds ratio of 0.16 (95% confidence interval 0.05–0.50) (Table 2, Supplementary Table S3). Due to high linkage disequilibrium between the two *SLC7A8* variants (R^2^ = 0.78), results were comparable for the C allele of rs8013529. In addition to the *SLC7A8* variants, three other variants (*CYP8B1* rs6771233, *SLC22A2* rs316003, *SLC22A5* rs274548) were significantly associated with EDP in the meta-analysis, showing a stronger association compared to the results of the discovery or validation cohorts alone.

In a meta-analysis including all samples, a significant association of *SLC7A8* rs1884545 with an odds ratio of 0.22 (95% confidence interval 0.07–0.63) and of *SLC7A8* rs8013529 with an odds ratio of 0.19 (95% confidence interval 0.06–0.55) was observed, corresponding to an approximately four to five-fold protective effect. Despite that, the association did not become stronger compared to the meta-analysis without these additional 27 patients (Table 2). The other variants, *SLC22A5* rs274548, *CYP8B1* rs6771233, and *SLC22A2* rs316003, remained significantly associated with EDP, with the latter two showing a stronger association.

### Validation of LAT2 overexpression model

**FIGURE 2:**
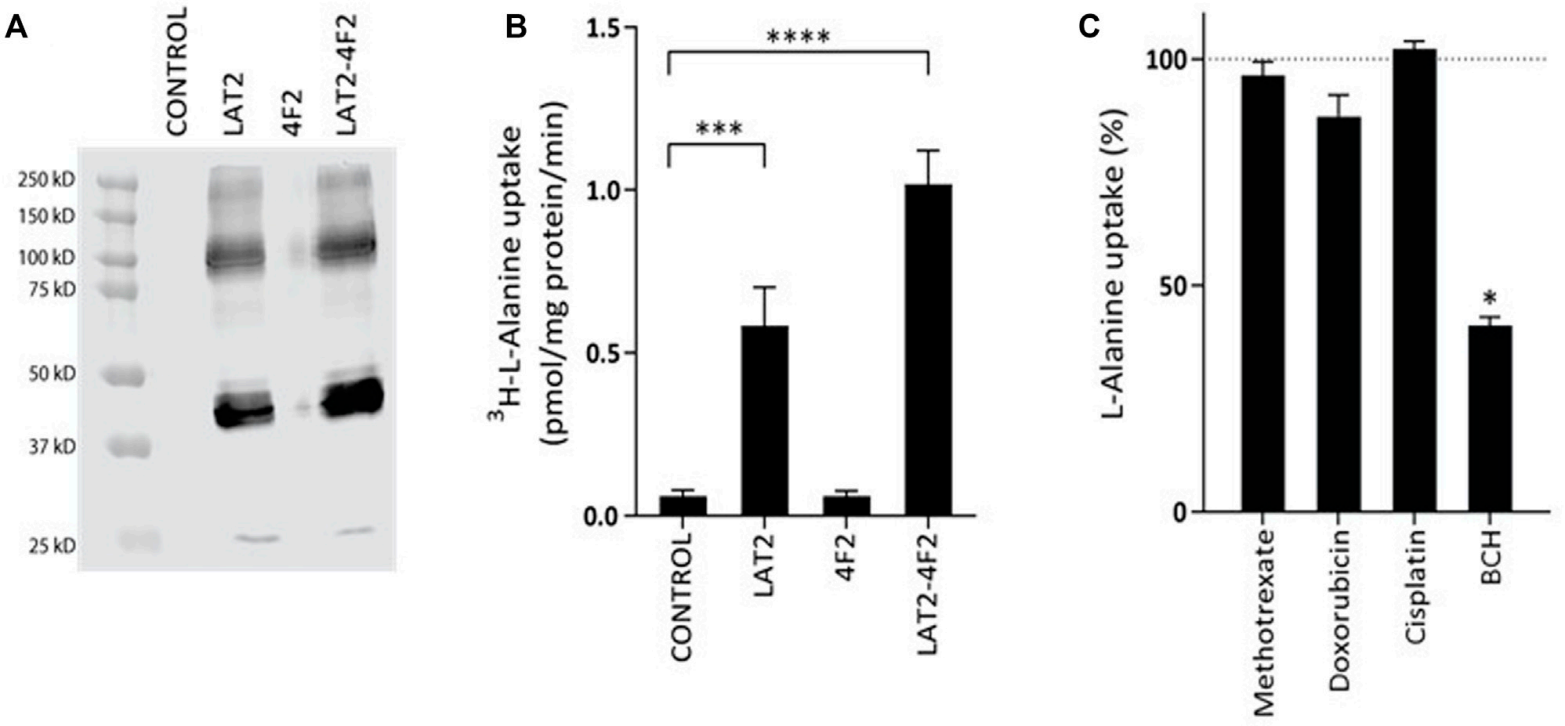
Western blot **(A)** and functional validation **(B,C)** of the transport assay in HEK293 cells overexpressing the L-type amino acid transporter 2 (LAT2). Figure A shows a western blot with the anti-LAT2 antibody. Bands are only visible in LAT2-HEK293 cells and LAT2-4F2-HEK293 cells. Figure B confirms ^3^H-L-alanine as a substrate of LAT2 and LAT2-4F2, after exposure to 0.018 µM of ^3^H-l-Alanine for 2 min at 37˚C. Data is expressed as mean ± SD (N = 3). Figure C shows L-alanine (10 µM) uptake, in the presence of cisplatin (1 mM), doxorubicin (1 mM), methotrexate (1 mM) or BCH (1 mM) , after 1 min incubation at 37˚C. BCH is a known inhibitor of LAT2-4F2 and was therefore included as a positive control. Inhibition was expressed as a percentage ± SD of L-alanine uptake with solvent control only, which was fixed at 100% (N = 3).

*SLC7A8* codes for the L-type amino acid transporter 2 (LAT2), which is most active when it is bound to its heterodimer 4F2. To study the role of the LAT2 transporter in chemotherapeutic treatment of osteosarcoma, an *in vitro* LAT2-4F2 overexpression cell model was developed. The Western blot with the anti-LAT2 antibody in Figure 2A only shows bands in the LAT2-HEK293 cells and the LAT2-4F2-HEK293 cells, indicating a LAT2 overexpression effect. Radiolabeled ^3^H-l-Alanine was used to functionally test and characterize LAT2-mediated transport in this model. Figure 2B shows that overexpression of LAT2 alone causes significant (*p* = 0.0001) uptake compared to control-transduced cells. It was confirmed that 4F2-HEK293 cells do not have any transport activity. Overexpression of both LAT2 and 4F2 further enhanced L-alanine uptake (*p* <0.0001).

To further characterize transport, time and concentration dependent uptake was studied in a time curve and Michaelis-Menten curve (Supplementary Figure S2). As shown in Supplementary Figure S2, the uptake rate is linear until two minutes, and therefore, the Michaelis-Menten curve was performed with an incubation time of one minute. The K_m_ for LAT2-4F2 mediated L-alanine uptake was estimated at 598 µM, with 95% confidence interval of 304–892 µM (Supplementary Figure S2).

### Interaction of LAT2-4F2 with cisplatin, doxorubicin and methotrexate

The association of a locus in the *SLC7A8* gene to EDP may be caused by an interaction of LAT2 with chemotherapeutics that are used in the treatment of osteosarcoma. First, potential inhibition of LAT2-4F2-mediated L-alanine transport was measured for cisplatin, doxorubicin and methotrexate. BCH (1 mM), a positive control inhibitor, significantly (*p* = 0.01) inhibited L-alanine uptake transport. Under the same conditions, cisplatin, doxorubicin or methotrexate did not significantly inhibit l-Alanine transport in this cell model (Figure 2C).

**FIGURE 3:**
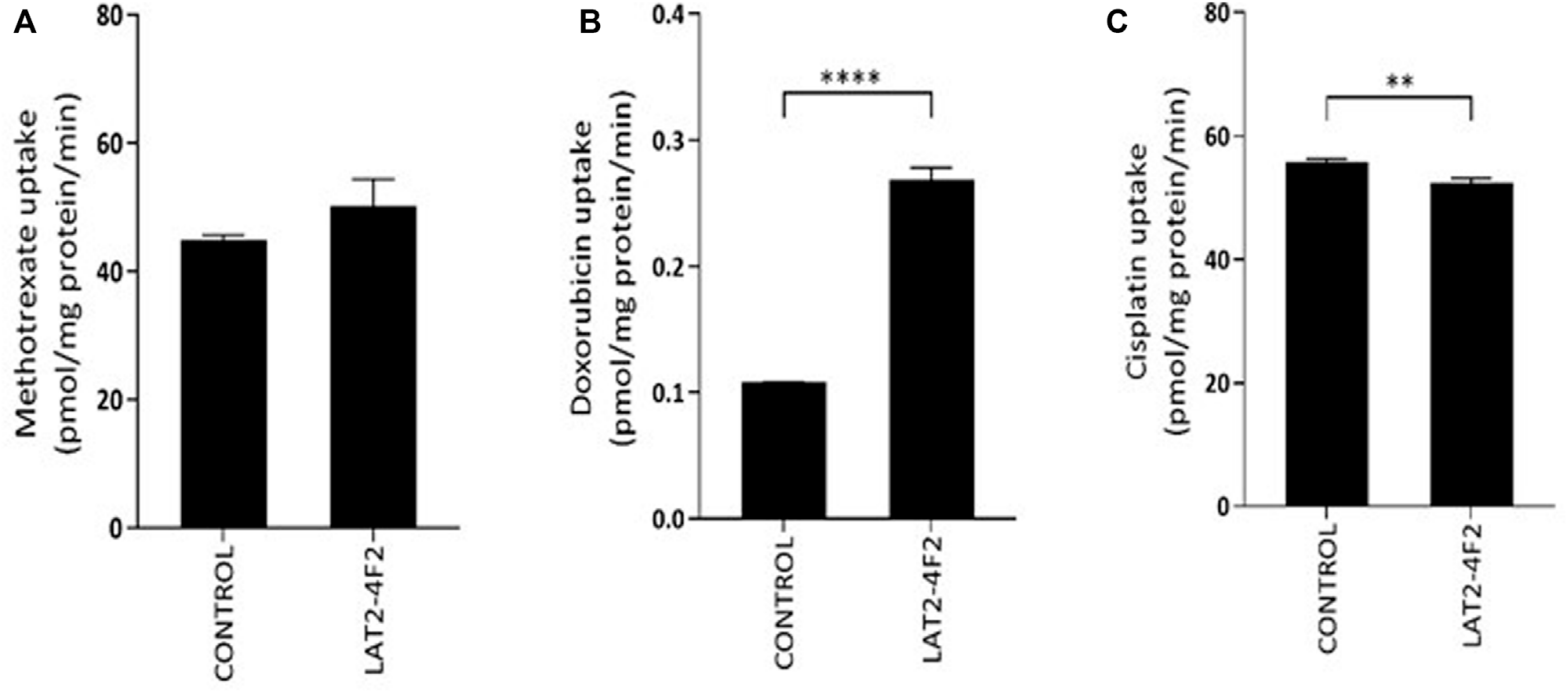
Transport assay to assess if methotrexate **(A)**, doxorubicin **(B)** or cisplatin **(C)** are substrates for LAT2-4F2. HEK293 cells were incubated with methotrexate (500 µM), doxorubicin (500 µM) and cisplatin (330 µM) for 30 min at 37˚C. No significant difference in uptake of methotrexate or cisplatin was found in LAT2-4F2-HEK293 cells (*p* = 0.1, *p* = 0.0028, resp.) compared control cells. Doxorubicin uptake was significant in LAT2-2F4-HEK293 cells (*p* <0.0001). All data is expressed as mean ± SD (N = 3).

Finally, transport assays were performed to assess if methotrexate, cisplatin or doxorubicin are substrates of LAT2-4F2 (Figure 3). No significantly increased uptake of ^3^H-methotrexate by LAT2-4F2 (*p* = 0.1) was found compared to the control. The accumulation of cisplatin was measured as platinum uptake by inductively coupled plasma mass spectrometry. As this technique was not previously used in this assay, a positive control transporter (OCT2) was also included. The uptake of cisplatin by OCT2 compared to control was significant (*p* < 0.0001, Supplementary Figure S3), but no uptake of cisplatin by LAT2-4F2 was measured. Finally, doxorubicin was identified as a substrate of LAT2-4F2 (*p* < 0.0001, Figure 3B) and transport of doxorubicin (10 µM) was inhibited to 81% (SD = 5.2%) by BCH (1 mM) under the same experimental conditions (*p* = 0.0032, *data not shown*). Altogether, doxorubicin, and not methotrexate or cisplatin, was shown to be a substrate for LAT2 which gives novel implications for the role of LAT2 in osteosarcoma therapy response to doxorubicin.

### mRNA expression analysis and immunohistochemistry

**FIGURE 4:**
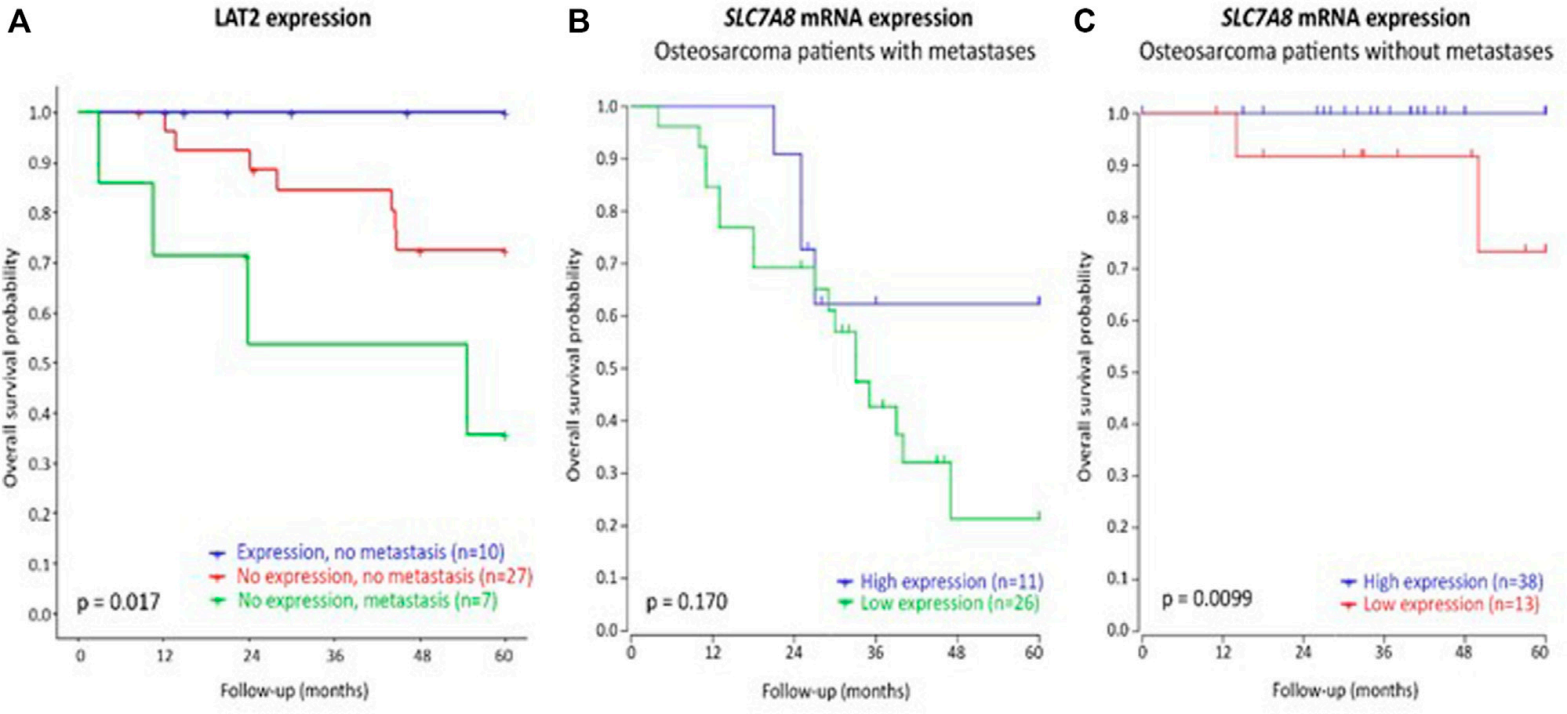
Overall survival of osteosarcoma patients according to or LAT2 protein expression **(A)**, stratified for the presence of metastasis, or *SLC7A8* mRNA expression **(B,C)**. Protein expression was determined by immunohistochemistry in the discovery cohort of this study and no patients with LAT2 expression and metastases at diagnosis were identified. mRNA expression was analyzed in an independent patient cohort (Kuijjer et al., 2012).

The presence of metastasis at diagnosis is the best clinical predictor of poor treatment outcome in patients with osteosarcoma, and therefore, protein expression analysis through immunohistochemistry and mRNA expression of osteosarcoma tissue were stratified for metastases. Figure 4A and Supplementary Figure S6 shows poorest overall survival and DFS, respectively, in patients with metastases at diagnosis and without LAT2 expression. Best DFS and overall survival was observed patients without metastases at diagnosis and with LAT2 expression (p_DFS_ = 0.025, p_OS_ = 0.017). This indicates that LAT2 expression may enhance survival, especially in patients without metastases at diagnosis. Similarly, *SLC7A8* mRNA expression is significantly associated with overall survival in patients without metastases (*p* = 0.0099, Figure 4C).

P-mTOR was stained in immunohistochemistry as mTOR phosphorylation was previously implicated to be a downstream process of LAT2-mediated amino-acid uptake (Kurayama et al., 2011; Wang and Holst, 2015; Feng et al., 2018). A representable example of LAT2 expression in tissue is displayed in Supplementary Figure S4. Supplementary Table S4 describes that LAT2 expression was present in 25.5% of patients and p-mTOR in 27.9% of patients. Expression of LAT2 or p-mTOR was not significantly associated with the *SLC7A8* rs1884545 T allele or EDP (Supplementary Table S5). In addition, LAT2 expression was not significantly associated with p-mTOR expression (OR (95%CI) = 3.5 (0.8–15.947), *p* = 0.117). Although not significant, p-mTOR expression showed a trend towards poorer DFS (p_DFS_ = 0.08, p_OS_ = 0.92, Supplementary Figure S5) and LAT2 expression may improve DFS and overall survival, however this was neither significant (p_DFS_ = 0.15, p_OS_ = 0.081, Supplementary Figure S5).

## Discussion

In our study we provided evidence that five genetic variants are associated with EDP in osteosarcoma, of which a locus in *SLC7A8* was confirmed in an independent validation cohort. Functional analysis showed that L-type amino acid transporter 2 (LAT2, gene *SLC7A8*) is involved in the transport of doxorubicin. Our results may have novel implications for personalized treatment of patients with osteosarcoma, because we suggest that LAT2-mediated doxorubicin uptake in osteosarcoma tumor cells could play an important role in treatment resistance and eventually treatment response.

In this study, we identified a novel locus in the *SLC7A8* gene to be associated with EDP in patients with osteosarcoma. The *SLC7A8* gene encodes the LAT2 transporter present in the basolateral membrane of the proximal tubule in the kidney, as well as in the colon and intestine (Rossier et al., 1999). LAT2 (light chain) is active when it forms a heterodimer with 4F2 (heavy chain) *via* a disulphide bond and is involved in the uptake and efflux of large and small neutral amino acids. At our current state of knowledge, little is known on the role and function of LAT2 in osteosarcoma or osteosarcoma treatment response, although it has been assessed in pharmacogenetic studies in other cancer types. Other germline variants and in this gene, that are not in linkage disequilibrium with the locus we identified, have been linked to outcome after platinum-based therapy in esophageal cancer (Rumiato et al., 2016). Acquired mutations in *SLC7A8* in the same patient group caused resistance to cisplatin-verapamil combination therapy and these results were reinforced by functional experiments (Min et al., 2021). When looking at expression of LAT2 in tumor tissues, LAT2 expression was associated with improved survival in estrogen receptor positive (ER+) breast cancer (Thakkar et al., 2015; El Ansari et al., 2020) and in lung cancer (Asada et al., 2020). EL Ansari *et al.* describes that *SLC7A8* mRNA expression and LAT2 protein expression in ER + breast cancer were strongly associated with good prognostic features. Conversely, LAT2 expression was associated with poorer survival in pancreatic cancer and was indicated to play a role in the pathogenesis of glomerulonephritis, both through LAT2-mediated mTOR activation (Kurayama et al., 2011; Feng et al., 2018).

Our results show that patients with expression of LAT2 in osteosarcoma tissue may have better survival compared to patients without expression of LAT2, especially in patients that present without metastases. Previous literature showed that amino acid uptake by LAT2 could enhance mTOR phosphorylation leading to mTORC1 activation and thereby a less favourable prognosis (Kurayama et al., 2011; Wang and Holst, 2015; Feng et al., 2018). Therefore, p-mTOR expression was assessed by immunohistochemistry. Based on this hypothesis it would be expected that high LAT2 expression is associated with p-mTOR and both would result in poorer overall survival (OS) and DFS. However, our non-significant results are in contrast with those expected if LAT2 led to mTORC1 phosphorylation suggesting no LAT2-mediated mTOR phosphorylation in tissue of osteosarcoma patients.

Apart from large and small neutral amino acids, other studies have shown that LAT2 also contributes to the transport of various medicines e.g., l-DOPA, melphalan, baclofen, gabapentin, and thyroid hormones (del Amo et al., 2008). However, a potential interaction between LAT2 and osteosarcoma chemotherapeutics has never been investigated. In this study, we found that doxorubicin is a Na^+^-independent substrate of LAT2. In combination with the association of LAT2 expression in tumor tissue with improved survival in patients without metastasis, LAT2-mediated doxorubicin transport could mechanistically explain the association of genetic variation in *SLC7A8* that is associated with EDP in patients with osteosarcoma. A previous study in neuroendocrine tumors showed that LAT1 and LAT2 expression in tumor cells caused increased l-DOPA uptake (Barollo et al., 2016). We hypothesize that increased LAT2 expression in osteosarcoma tissue could lead to increased uptake of doxorubicin, therefore higher intracellular exposure and improved cell death. Future research should further address this to translate the findings of this study to the clinical setting.

The genetic variants in the *SLC7A8* locus associated with EDP include a synonymous variant (rs1884545) and an intron variant (rs8013529). The exact effect of these variants on the protein function or on protein expression remains unknown. Additional studies, for example through fine-mapping or functional experiments are necessary to give additional insight.

Notably, two other genes (*SLC22A2,* Solute carrier family 22 member 2 and *SLC22A5,* Solute carrier family 22 member 5) of the four identified genes also encode transporters that function in the kidneys. *SLC22A2*, which codes for the organic cation transporter 2 (OCT2) is implicated in the transport of cisplatin in tubular cells, which was also confirmed in this study (Supplementary Figure S3). A genetic variant in the gene (rs316019, not in LD with rs316003) has been linked to cisplatin induced nephrotoxicity (Filipski et al., 2009; Iwata et al., 2012; Zhang and Zhou, 2012). *SLC22A5* is involved in the reabsorption of carnitine by the proximal tubular cells. Studies have indicated that cisplatin inhibits *SLC22A5* functioning also leading to nephrotoxicity (Haschke et al., 2010; Lancaster et al., 2010). The fourth identified gene (*CYP8B1,* Cytochrome P450 8B1) is expressed in hepatocytes and is involved in bile acid production and glucose homeostasis (Kaur et al., 2015). Thus far, no clear connection between this gene and chemotherapy treatment has been reported.

We have pharmacogenetically studied the largest cohort of osteosarcoma patients with EDP to date and are the first to include independent validation cohorts. Nevertheless, patient numbers are small for genetic association studies. This however reflects the rarity of the disease and even for these numbers, international collaboration was required. Therefore, we consider the current study as a first but important step into the pharmacogenetic background of a suboptimal drug response in patients with osteosarcoma. As we have retrospectively included patients diagnosed over the past decades, during which imaging techniques have improved, it is possible that we have missed some cases of EDP in patients diagnosed in the early years. In addition, the discovery cohort was heterogeneous regarding treatment protocols. A proportion of the patients received two drugs (doxorubicin and cisplatin), whereas others received drugs in addition to cisplatin and doxorubicin (mostly only MTX), which could give a more favorable outcome. However, there was no significant effect of the presence of MTX in the treatment regimen on EDP in our cohort, which makes it likely that the influence of the differences in treatment regimens on the results is limited. Furthermore, because we studied germline variants, we may have missed tumor-specific mutations in genes involved in the uptake of the chemotherapeutic drugs, although studies on genetic variants in genes involved in drug metabolism and transport showed high concordance between DNA derived from tumor and blood or saliva (van Huis-Tanja et al., 2013). In addition, the tumor genetic background is also likely to define the intrinsic response to chemotherapy (Kuijjer et al., 2013).

The goal of identifying pharmacogenetic variants involved in EDP is to enable pre-treatment identification of patients who are at pharmacogenetic risk of a poor response to treatment with conventional chemotherapy. The genetic loci identified in the present study are not yet discriminative for implementation in the clinical setting, but the evidence that is presented here does present novel opportunities for the future. In addition, it is to be expected that treatment outcome is not determined by one gene, therefore, in the future it is to be expected that combination of genes will be used to identify patients at risk of poor response to treatment. Upfront identification of such patients could provide an opportunity to personalize therapy and help achieve a better balance of treatment outcome and toxicity burden. For example, other second-line treatments could be considered as first-line treatment for this subgroup of patients (Harris et al., 1995; Rodriguez-Galindo et al., 2002; Navid et al., 2008; Berger et al., 2009; Song et al., 2014; Grignani et al., 2015). In addition to such a clinical implementation, these genetic association studies are important to gain more insight into the mechanisms of action of the drugs investigated.

## Data Availability

The datasets presented in this study can be found in online repositories. The names of the repository/repositories and accession number(s) can be found below: https://easy.dans.knaw.nl/ui/datasets/id/easy-dataset:256317, https://doi.org/10.17026/dans-2b8-yswb.
