# Supplementary material for: SLC7A8 coding for LAT2 is associated with early disease progression in osteosarcoma and transports doxorubicin
Source: Front Pharmacol. 2022 Nov 9;13:1042989. doi: 10.3389/fphar.2022.1042989 (PMC9681801; doi:10.3389/fphar.2022.1042989)
Supplement: Supplementary file 1 [file DataSheet1.DOCX]

**Supplementary data**

**
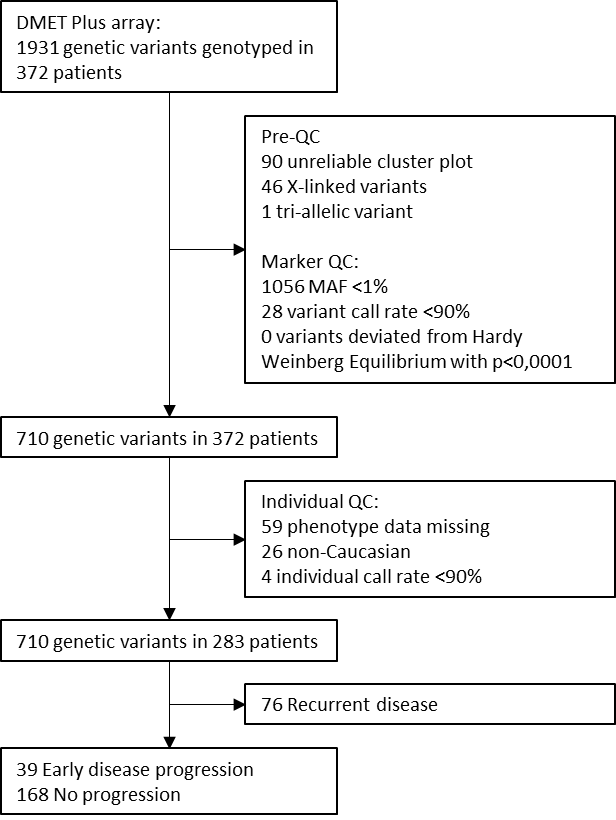
**

**Figure S1. Flow chart of quality control (QC).**

**Table S1.** **Clinical characteristics of osteosarcoma patients of the discovery cohort**, including patients without sufficient DNA for DMET array genotyping, but enough for manual genotyping of genetic variants significantly associated to progressive disease in the original patient cohort. As this mostly concerns deceased patients, it is critical to involve this group that was not randomly missing.

|  | **Original discovery cohort** | | **Additional patients with limited DNA** | | **Total cohort** | |
| --- | --- | --- | --- | --- | --- | --- |
|  | **Progression (*N*=39)** | **No progression (*N*=168)** | **Progression (*N*=11)** | **No progression (*N*=5)** | **Progression (*N*=50)** | **No progression (*N*=173)** |
| Age at diagnosis, median (range) | 17.2 (6.8-44.7) | 15.2 (3.4-45.8) | 17.7 (2.8-34.1) | 19.9 (14.6-44.5) | 17.2 (2.8-44.7) | 15.3 (3.4-45.8) |
| Male sex, *n* (%) | 29 (74.4%) | 81 (48.2%) | 8 (72.7%) | 3 (60%) | 37 (74%) | 84 (48.6%) |
| Axial tumor, *n* (%) | 4 (10.3%) | 5 (2.98%) | 2 (18.2%) | 2 (40%) | 6 (12%) | 7 (4%) |
| Primary metastases, *n* (%) | 16 (41.0%) | 16 (9.52%) | 4 (36.4%) | 2 (40%) | 20 (40%) | 18 (10.4%) |
| Cumulative dose (mg/m^2^)  Cisplatin^a^, median (range)  Doxorubicin, median (range) | 400 (100-600)  450 (150-450) | 480 (200-720)  450 (150-455) | 300 (200-600)  450 (420-550) | 540 (260-600)  450 (405-450) | 400 (100-600)  450 (150-550) | 480 (200-720)  450 (150-455) |
| MTX treatment, *n* (%) | 27 (69.2%) | 90 (53.6%) | 3 (27.3%) | 1 (20%) | 40 (60%) | 91 (52.6%) |
| Poor histologic response^b^, *n* (%) | 28 (82.4%) | 78 (48.8%) | 6 (100%) | 1 (50%) | 34 (85%) | 79 (48.5%) |
| 5-year overall survival | 28.2% | 100% | 0% | 60% | 22% | 98.9% |

MTX, methotrexate

Patients with recurrent disease are not included in the table: discovery cohort *N*=76; additional cohort *N*=9.

^a^Number of patients with cumulative dose of cisplatin data available; discovery cohort: progression *N*=39, controls *N*=165; additional cohort: all patients

^b^Number of patients with histologic response data available; discovery cohort: progression *N*=34, controls *N*=160; additional cohort: progression *N*=6, controls *N*=2

**Table S2**. **Association analysis results for the discovery cohort,** including 16 additional non-randomly excluded patients due to limited availability of DNA.

| **SNP** | **Gene** | **Chromosome** | **Minor allele** | **MAF** | **OR** | **95% CI** | ***P*-value** |
| --- | --- | --- | --- | --- | --- | --- | --- |
| rs1884545 | *SLC7A8* | 14 | T | 0.12 | 0.27 | 0.1-0.77 | 0.014 |
| rs8013529 | *SLC7A8* | 14 | C | 0.12 | 0.29 | 0.1-0.79 | 0.016 |
| rs6771233 | *CYP8B1* | 3 | C | 0.34 | 1.51 | 0.89-2.56 | 0.129 |
| rs316003 | *SLC22A2* | 6 | G | 0.21 | 0.52 | 0.26-1.06 | 0.073 |
| rs274548 | *SLC22A5* | 5 | T | 0.14 | 1.62 | 0.81-3.25 | 0.170 |

MAF, minor allele frequency; OR, odds ratio; 95% CI, 95% confidence interval.

OR and 95% CI are reported for the minor allele, an OR<1 indicates a protective effect of the minor allele and risk of progression for the major allele.

**Table S3.** **Association analysis results validation cohort and meta-analyses**, including 16 patients with limited DNA available in discovery cohort.

|  |  |  |  | **Validation** | | | | | | **Second validation** | | |
| --- | --- | --- | --- | --- | --- | --- | --- | --- | --- | --- | --- | --- |
|  |  |  |  | **Validation cohort** | | | **Meta-analysis** | | | **Meta-analysis overall** | | |
| **SNP** | **Gene** | **Chromosome** | **Minor allele** | **OR** | **95% CI** | ***P*-value** | **OR** | **95% CI** | ***P*-value** | **OR** | **95% CI** | ***P*-value** |
| rs8013529 | *SLC7A8* | 14 | C | 0.05 | 0.005-0.63 | 0.02 | 0.21 | 0.08-0.55 | 0.001 | 0.24 | 0.09-0.59 | 0.002 |
| rs1884545 | *SLC7A8* | 14 | T | 0.05 | 0.005-0.63 | 0.02 | 0.22 | 0.09-0.57 | 0.002 | 0.26 | 0.1-0.65 | 0.004 |
| rs6771233 | *CYP8B1* | 3 | A | 1.7 | 0.78-3.72 | 0.184 | 1.57 | 1.01-2.43 | 0.045 | 1.61 | 1.05-2.49 | 0.03 |
| rs316003 | *SLC22A2* | 6 | G | 0.58 | 0.20-1.69 | 0.318 | 0.54 | 0.3-0.97 | 0.041 | 0.54 | 0.31-0.96 | 0.035 |
| rs274548 | *SLC22A5* | 5 | T | 1.75 | 0.59-5.18 | 0.311 | 1.66 | 0.93-2.98 | 0.089 | 1.64 | 0.93-2.89 | 0.086 |

OR, odds ratio; 95% CI, 95% confidence interval; NA, not analyzed.

OR and 95% CI are reported for the minor allele, i.e. an OR<1 indicates risk of progression for the major allele.

^a^ I^2^>50.


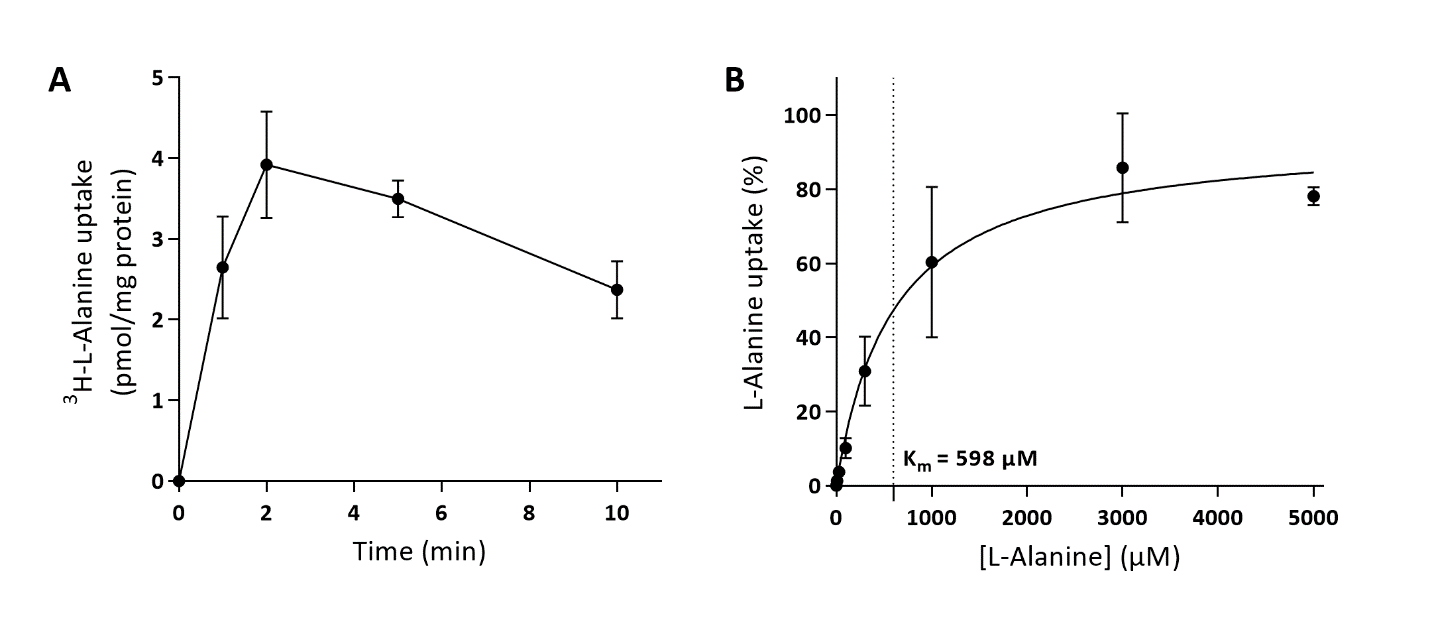


**Figure S2 Time- and concentration dependent uptake of L-Alanine in LAT2-4F2-HEK293 cells**. Figure **A** shows ^3^H-L-Alanine uptake in LAT2-4F2-HEK293 cells after exposure of 0.017 µM ^3^H-L-Alanine at 37˚C. The uptake rate is linear until 2 minutes, and therefore, the Michaelis-Menten curve was performed with 1 minute incubation time. For **B**, cells were exposed to increasing concentrations of L-Alanine and 0.017 µM ^3^H-L-Alanine. The K_m_ for LAT2-4F2 mediated L-alanine uptake was estimated at 598 µM (95% CI = 304 µM – 892 µM). All signals were subtracted by the background signal that was measured in EYFP-HEK293 cells under the same conditions. Data in figure A is expressed as mean ± SD (N=3). Figure B is expressed as a percentage ± SD of V_max_, which was fixed at 100% (N=3).


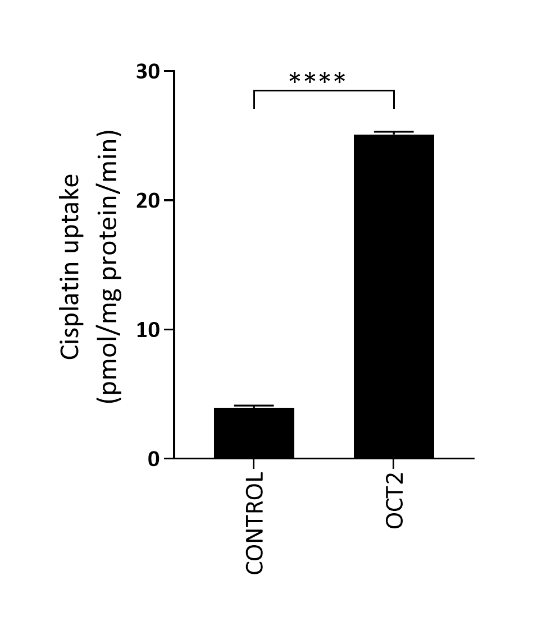


**Figure S3 Transport assay to validate that platinum concentration measurements by ICP-MS are appropriate to measure uptake in this cell model.** The *SLC22A2* gene codes for the organic cation transporter 2 (OCT2), which was previously indicated in the transport of cisplatin. 50 µM cisplatin was exposed for 10 minutes to EYFP-HEK293 cells and LAT2-4F2-HEK293 cells at 37˚C. The cisplatin uptake was significantly higher in LAT2-2F4-HEK293 cells (p < 0.0001). Altogether, it was concluded that cisplatin uptake was measured accordingly by ICP-MS. All data is expressed as mean ± SD (N=3).


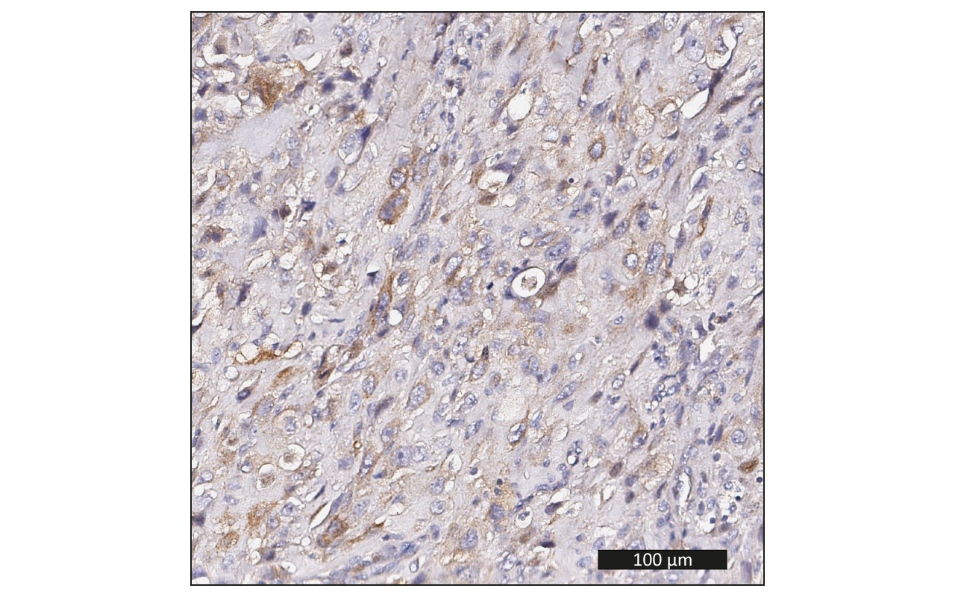


**Figure S4. Representative example of LAT2 protein expression in osteosarcoma tissue at diagnosis.** Brown staining indicates LAT2.

**Table S4. Immunohistochemistry staining of LAT2 and p-mTOR in osteosarcoma tissue at diagnosis**. Patients with <10% expression are considered controls and patients with ≥10% expression are considered cases.

|  | **LAT2 expression** | | | **p-mTOR expression** | | |
| --- | --- | --- | --- | --- | --- | --- |
|  | All patients  (N=47) | Progressive disease^a^  (N=7) | No progressive disease  (N=26) | All patients  (N=43) | Progressive disease^a^  (N=7) | No progressive disease  (N=23) |
| Controls | 35 (74.5%) | 7 (100%) | 17 (65.4%) | 31 (72.1%) | 4 (57.1%) | 20 (87%) |
| Cases | 12 (25.5%) | 0 (0%) | 9 (34.6%) | 12 (27.9%) | 3 (42.9%) | 3 (13%) |

^a^ Progressive disease was defined as: (1) growth of the primary tumor (>20%) and/or metastases (>20%), or development of new lesions, in the time from start of primary treatment until 3 months after end of adjuvant chemotherapy or end of first-line treatment in case of primary metastatic disease, and/or (2) inadequacy to reach complete remission at the end of (surgical and chemotherapeutic) therapy for primary localized or primary metastatic osteosarcoma. The opposite extremes, patients showing an adequate drug response with no signs of relapse were considered controls. Thus patients with recurrent disease, defined as local or distant relapse from 3 months after end of primary treatment to end of follow-up, were excluded from the comparison for progressive disease.

**Table S5. Association of LAT2 or p-mTOR protein expression with genetic variant or disease progression**, calculated with X^2^ test.

|  | **LAT2 expression** | | | **p-mTOR expression** | | |
| --- | --- | --- | --- | --- | --- | --- |
|  | N | OR (95%CI) | *p* | N | OR (95%CI) | *p* |
| *SLC7A8* rs1884545 T-allele | 47 | 0.26 (0.03-2.33) | 0.414 | 43 | 1.71 (0.40-7.43) | 0.467 |
| Progressive disease | 33 | 0.65 (0.49-0.87) | 0.149 | 30 | 5 (0.73-34.35) | 0.361 |


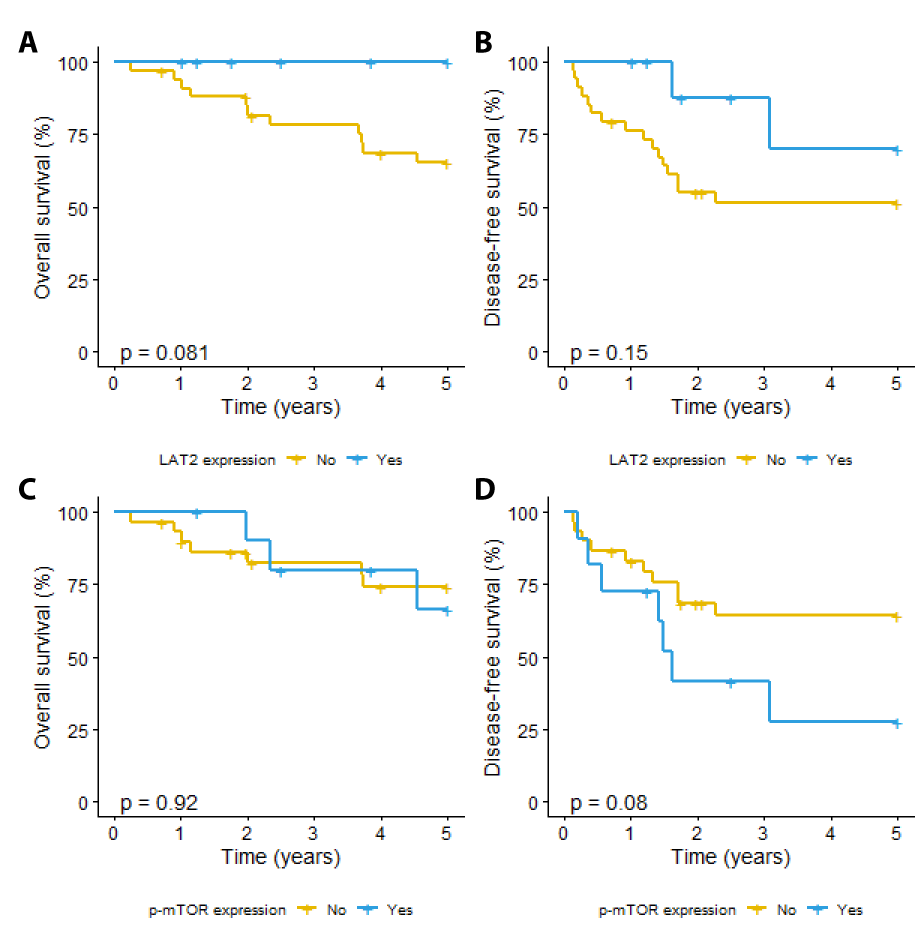


**Figure S5. Kaplan Meier survival curves of overall survival (A and C) and disease-free survival (B and D)** in osteosarcoma patients with and without LAT2 (A and B) or p-mTOR (C and D) protein expression in tumor tissue at diagnosis. P-values are the result of a log-rank test.


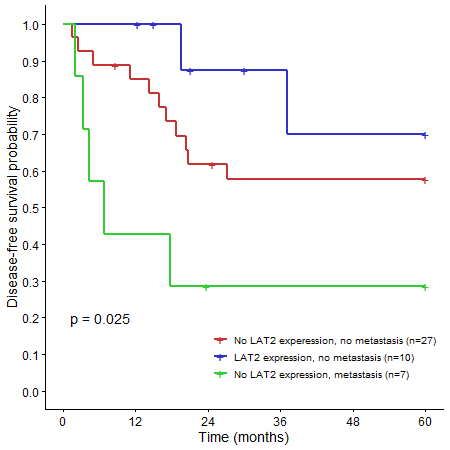


**Figure S6. Kaplan Meier survival curve of disease-free survival of osteosarcoma patients stratified for LAT2 expression in osteosarcoma tissue and metastasis at diagnosis.** In this cohort, no patients with LAT2 expression and metastases at diagnosis were identified.
